# Multilayer network based comparative document analysis (MUNCoDA)

**DOI:** 10.1016/j.mex.2020.100902

**Published:** 2020-04-29

**Authors:** Viktor Sebestyén, Endre Domokos, János Abonyi

**Affiliations:** a University of Pannonia, MTA-PE "Lendület" Complex Systems Monitoring Research Group; b University of Pannonia, Institute of Environmental Engineering

**Keywords:** Text-mining, Multi-document summarization, Document clustering, Network similarity

## Abstract

The proposed multilayer network-based comparative document analysis (MUNCoDA) method supports the identification of the common points of a set of documents, which deal with the same subject area. As documents are transformed into networks of informative word-pairs, the collection of documents form a multilayer network that allows the comparative evaluation of the texts. The multilayer network can be visualized and analyzed to highlight how the texts are structured. The topics of the documents can be clustered based on the developed similarity measures. By exploring the network centralities, topic importance values can be assigned. The method is fully automated by KNIME preprocessing tools and MATLAB/Octave code.

• Networks can be formed based on informative word pairs of a multiple documents
• The analysis of the proposed multilayer networks provides information for multi-document summarization
• Words and documents can be clustered based on node similarity and edge overlap measures

## Graphical abstract

**Figure:**
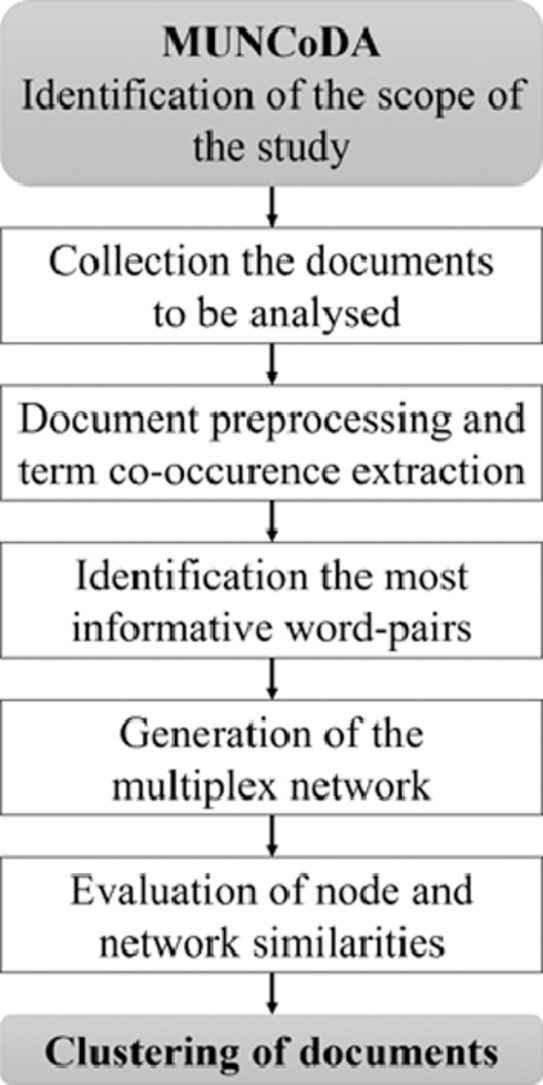

Specifications table

**Table:** 

Subject Area:	Computer Science
More specific subject area:	*Network analysis and text mining*
Method name:	*Multilayer network based comparative document analysis (MUNCoDA) method*
Name and reference of original method:	*Term frequency-inverse document frequency:* Robertson, Stephen. "Understanding inverse document frequency: on theoretical arguments for IDF." Journal of documentation (2004). *Multilayer networks:* Boccaletti, Stefano, et al. "The structure and dynamics of multilayer networks." Physics Reports 544.1 (2014): 1-122.
Resource availability:	https://github.com/abonyilab

## Method details

Multi-document summarization extracts information from multiple texts written about the same topic. The purpose of the method is to objectively explore the relationships between documents, identify key topics and compare documents according to the explored set of focal points. Contrary to classical multi-document summarization the extracted information is represented in a multiplex network which layers represent the network of the most informative word-pairs of the documents. The automated analysis of the network provides objective information about the topics and similarities of the documents.

**Fig. 1:**
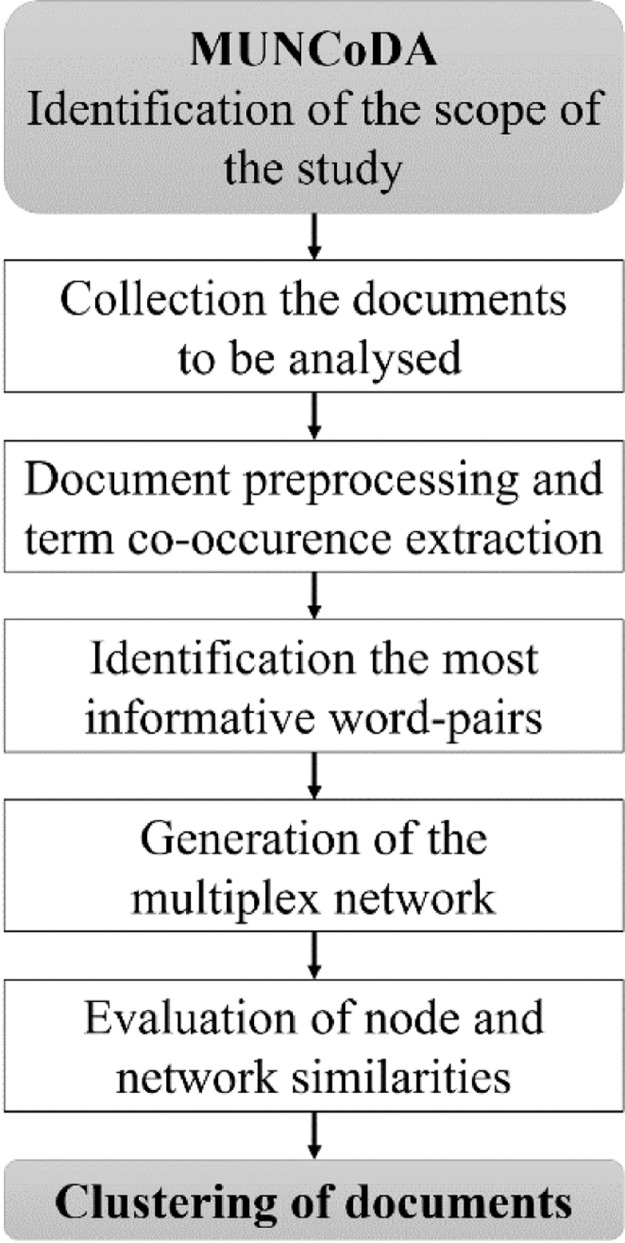
The process of the proposed multilayer network-based comparative document analysis method.

The steps of the proposed method are shown in Fig. 1. In the first step, the scope of the analysis needs to be identified that determines which documents are available for the study of the topic (e.g., sustainability reports, scientific papers, etc.). The next step is the preprocessing of the text that means the removal of stopwords, stemming, removal of short/long terms, and removal of frequent/infrequent terms (locally or globally) and term weighting and normalization. The informative word pairs are extracted from the word co-occurrences define the edges of the networks of the documents. Finally, the multiplex network is generated where the layers represent the networks of the documents. In the final step, the nodes and the layers are clustered based on their similarities.

### Background on MUNCoDA algorithm

Text mining is becoming a frequently utilized tool to extract informative patterns from unstructured or semi-structured data sources [1]. Text mining can also help in Comparative Document Analysis (CDA), which means the joint discovery of commonalities and differences between two individual documents [2]. Multi-document summarization aims to produce a summary delivering the majority of information content from a set of documents about an explicit or implicit main topic [3]. The result of multi-document analysis allows the comparative evaluation of document content. Semantic similarity of words and documents can be determined by human decision-based, information content-based [4], probability-based or word-pair-based [5] approaches.

Network analysis techniques may be used to understand better the relationships between texts, based on which quantitative (e.g., degree, centrality, clustering coefficient) and qualitative semantic interpretations (e.g., collocation, semantic relation, encoding semantic topics) can be made [6]. Ontology-based approaches, which use advanced text mining tools to unlock the inherent relationship between concepts, can be used to improve the retrieval of design information in a large, unstructured text data [7]. With networks not only the relationships within a document be discovered, but also whether there is overlap between information from different sources [8]. Text mining combined with network analysis can also be used to extract keywords from various sources and track the evolution of a topic over time [9].

Informative word pairs of documents can form multilayer networks that are ideal models for representing multidimensional data [10]. The proposed method is based on the novel combination of the previously presented concept as it will be shown in the following subsections.

### Document preprocessing and term co-occurrence extraction

**Fig. 2:**
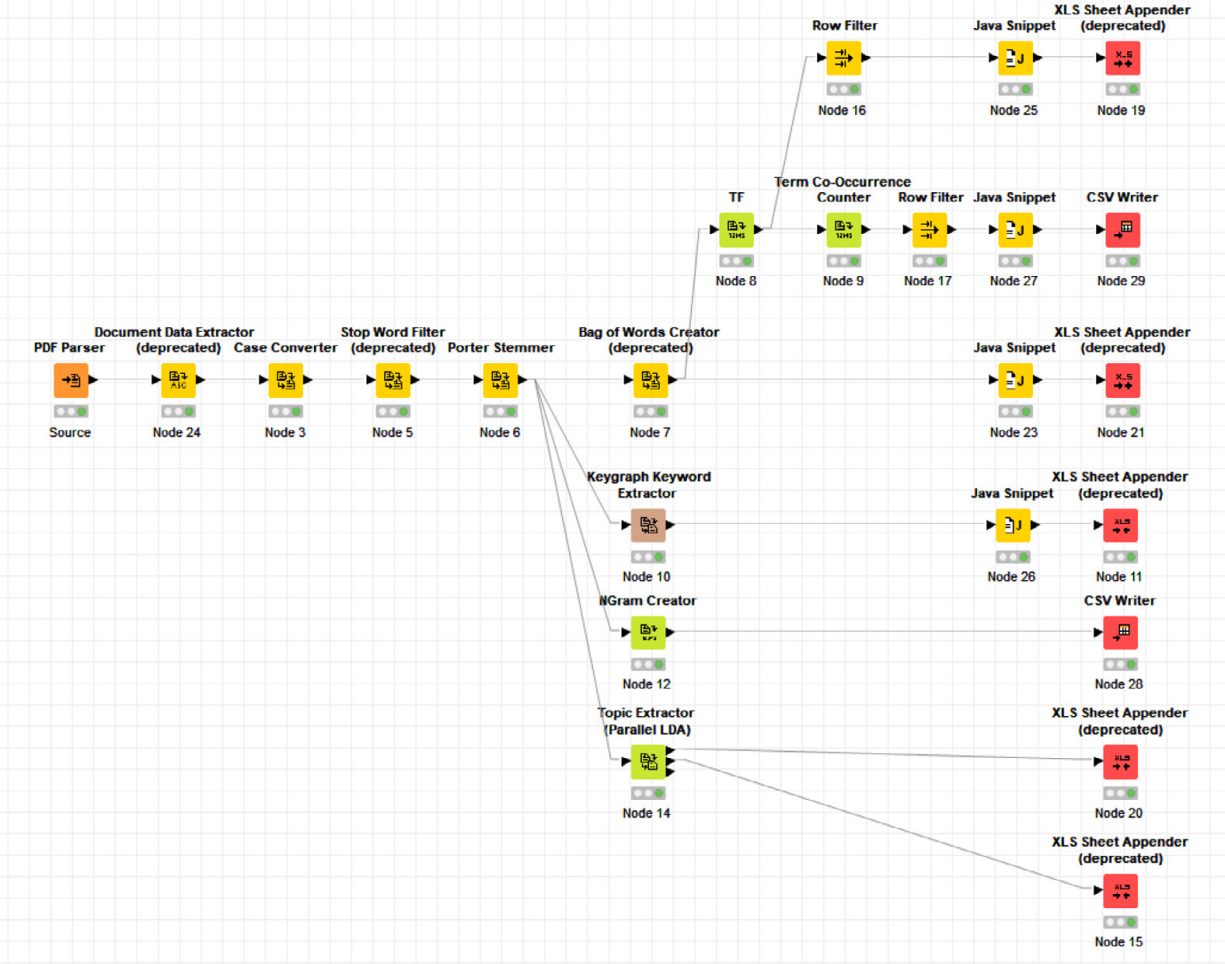
The preprocessing workflow developed in KNIME.

The first step of the KNIME workflow developed for the preprocessing of the documents is the text extraction from the pdf files (see Fig. 2.). After the extraction of document-related information and conversion the text to lower case, the stop words are eliminated. A Porter Stammer node is used for stemming, which means the reduction of the inflectional and derivationally related forms of a word to a common base form. The method processes the bag of words containing the terms occurring in the documents. The TF node computes the relative term frequency (tf) of each term according to each document and adds a column containing the tf value. The Term Co-Occurrence counter node counts the number of co-occurrences for the given list of terms within the selected parts e.g. sentence, paragraph, section and title of the corresponding document. At the end of the text mining, the processed data is written to excel or csv files.

Besides the core preprocessing process, the relevant keywords are also extracted by a graph-based approach (with the Keygraph Keyword Extractor node), NGrams were also generated by the and the NGram Creator node. The Topic Extractor node has also been utilized to cluster the words based on the latent Dirichlet allocation (LDA) generative statistical model which topic areas are used for verification.

### Extraction of the most informative word-pairs

Contrary to classical text mining, besides studying the frequencies of individual words, the distributions of word pairs in the documents were considered, so in this method the word pairs are referred as terms, denoted by *t*.

To measure how commonly or rarely the term is applied across all documents, the number of documents containing the terms *n_t_* is calculated [11]:

(1) $$n_{t}=\left|\left\{d\in D:t\in d\right\}\right|$$

where *D* represents the set of the studied documents, so *n_t_* is between 1 and M=|D|, where *M* stands for the number of documents.

As the *P*(*t*|*D*) conditional probability represents how common or rare is the term across the documents, the logarithmically scaled inverse fraction of the documents that contain a term is used to evaluate how much information the term provides.:

(2) $$idf\left(t,D\right)=-\log P\left(t|D\right)=-log\frac{n_{t}}{M}=log\frac{M}{n_{t}}$$

The occurrence of the word pairs in a given document are represented by the variable *f_t,d_*, that shows how many times a term *t* occurs in a given document *d*. To prevent bias towards longer documents, these *f_t,d_* raw term counts are normalized by the raw count of the most frequently occurring terms in the document, so the augmented frequency is calculated as:

(3) $$tf\left(t,d\right)=K+\left(1-K\right)\frac{f_{t,d}}{max_{t^{\prime }\in d\;ft^{\prime },d}},\;if\;f_{t,d}>0$$

where *K=[0,1]* is a tuning parameter, usually *K=0.5*;

The “term frequency - inverse document frequency” measure combines the previously presented term frequency and inverse document frequency measures to evaluate the information content of a term:

(4) tfidf(t,d,D)=tf(t,d)idf(t,D)

Based on the presented measures the extracted word-pairs are filtered as the word-pairs should appear in almost every documents, *n_t_* > *tr_n_*, and should provide useful information *tfidf*(*t, d, D*) > *tr_info_*.

### Generation of a multiplex network of documents

Our key idea is that the filtered the word-pairs can be used to form networks of the documents. As the *t* term consist of the pair of the *w_i_* -th and the,*w_j_*-th words, the edge between these words in the *d*-th layer of the multiplex network is weighted as $a_{i,j}^{d}=$ *tf*(*t, d*)|*w_i_, w_j_* ∈ *d*. In this way, a single network can be created for each document, from which a multiplex network can be defined.

In the resulted multiplex network, the nodes represent words, while the edges represent the word-pairs weighted by how frequent of their connection in the given document. The network has precisely as many layers as documents are analyzed (*M*) and consist of a fixed set of nodes (words) i,j=1,…,N connected by different types of links in each layer (where *N* represents the number of selected words from the *D* set of documents).

A general/global picture about the problem, which discussed in the input documents can be obtained by merging the layers of the multiplex network into a “total projection” layer. Instead of the aggregation of the layers [12], this layer is defined based on the average or the maximum of the *tf*(*t, d*) values,

(5) $$a_{i,j}^{*}=\frac{1}{M}\sum_{d=1}^{M}tf\left(t,d\right)|i,j\in d$$

(6) $$a_{i,j}^{+}=\max_{d}tf\left(t,d\right)|i,j\in d$$

### Evaluation of word and document similarities

The analysis is done by determining the degree of similarity between the nodes (words) and layers (documents) and evaluating the node centralities.

The similarity of words in a given document evaluated based on the relative term-frequency of the word-pairs, $s^{d}\left(w_{i},w_{j}\right)=tf\left(t,d\right)|w_{i},w_{j}\in d$, which is directly represented by the $a_{i,j}^{d}$ edge weighs. Based on this interpretation only similarities of the frequently co-occurring words are given. Based on the calculation of the $s^{d}\left(w_{i},w_{k}\right)=s^{d}\left(w_{i},w_{j}\right)s^{d}\left(w_{j},w_{k}\right)$ transitive similarities of the paths can be determined for each word-pairs. For the non-neighbor nodes the products of sequentially multiplying similarity values of node pairs of the paths can then be calculated.

As more than one path can exists among these paths, we select that has the minimum similarity degree, therefore the calculation of the transitive similarity leads to the problem of finding the *P* shortest path set [13]

(7) $$s^{d}\left(w_{i},w_{j}\right)=\min_{P\in P^{\prime }}\prod \left(w_{m},w_{r}\right)\in P^{\prime }s^{d}\left(w_{m},w_{r}\right)$$

The procedure can be easily implemented by the well-known Floyd's algorithm:

**Table:** 

for *k =1:N*
for *i =1:N*
for *j =1:N*
if $s^{d}\left(w_{i},w_{j}\right)$ < $s^{d}\left(w_{j},w_{k}\right)$$s^{d}\left(w_{k},w_{j}\right)$
$s^{d}\left(w_{i},w_{j}\right)$ ← $s^{d}\left(w_{j},w_{k}\right)$$s^{d}\left(w_{k},w_{j}\right)$
end if

The resulted $S^{d}={\left[s^{d}\left(w_{i},w_{j}\right)\right]}_{N\times N}$ similarity matrix of the *d*-th document allows the clustering the words in form of dendrograms and layer wise two-dimensional distance-preserving visualization of the words by multidimensional scaling (MDS), where the distance is $dist^{d}\left(w_{i},w_{j}\right)=1-s^{d}\left(w_{i},w_{j}\right)$. By using MDS, the subject areas do not need to be preliminary identified, the tool allows the objective identification of the subject areas. In a targeted study, when subject areas are known, it is possible to determine which words are most closely related to a particular expression (topic title), thus allows the measurement the depth of a document's coverage of the given subject. and clustering the thematic areas of the documents [14].

The similarity of the documents can be evaluated based on the aggregation of the pairwise comparison of the word similarities:

(8) $$L_{s}\left(\alpha ,\beta \right)=\sum_{i,j}\frac{min(s^{\alpha }\left(w_{i},w_{j}\right),s^{\beta }\left(w_{i},w_{j}\right)}{max(s^{\alpha }\left(w_{i},w_{j}\right),s^{\beta }\left(w_{i},w_{j}\right)}$$

The above presented *L_s_*(*α, β*) measure can be considered as the transitive similarity based extension of the edge overlap measure that is used to compare the similarities of the layers of multiplex networks [14]. The developed measure can be also used to evaluate the coverage of a given document: as $c\left(d\right)=L_{s}\left(d,+\right)$ measures how the *d* document is similar to the layer defined based the maximum of the tf(t,d) values.

The importance of the words and word-pairs can be evaluated based on classical network centrality measures. As these measures are well known and available in most of the network analysis packages, we do not discuss them in this paper. We only note that the documents can also be compared based on the rank correlation of these measures.

The calculation of the similarities has been implemented in MATLAB and the code is available at the website of the corresponding author (https://github.com/abonyilab/MUNCoDA).

## Method validation

The methodology is validated based on Voluntary National Reviews (VNR) [15]. The VNRs of 75 countries were analyzed, which were available in English. In this complex example, MUNCAD's method has been used to explore potential future collaboration opportunities for the countries and to identify topic areas of the implementation of 2030 Agenda.

**Fig. 3:**
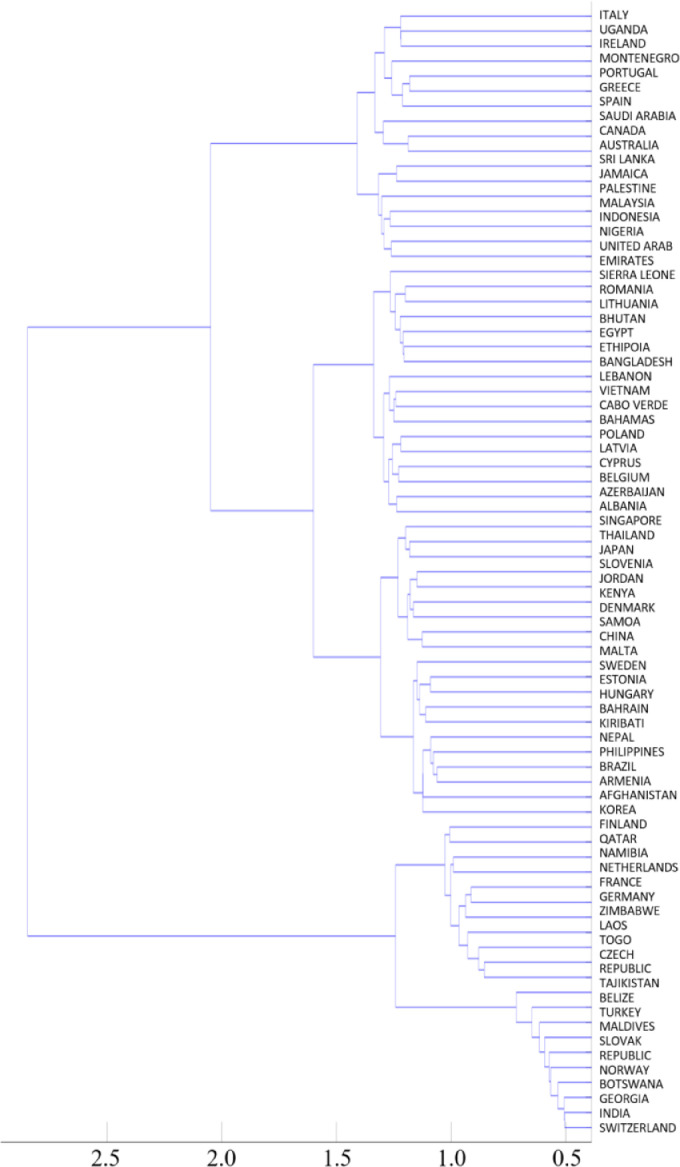
The dendrogram-based clustering of the analyzed countries.

**Fig. 4:**
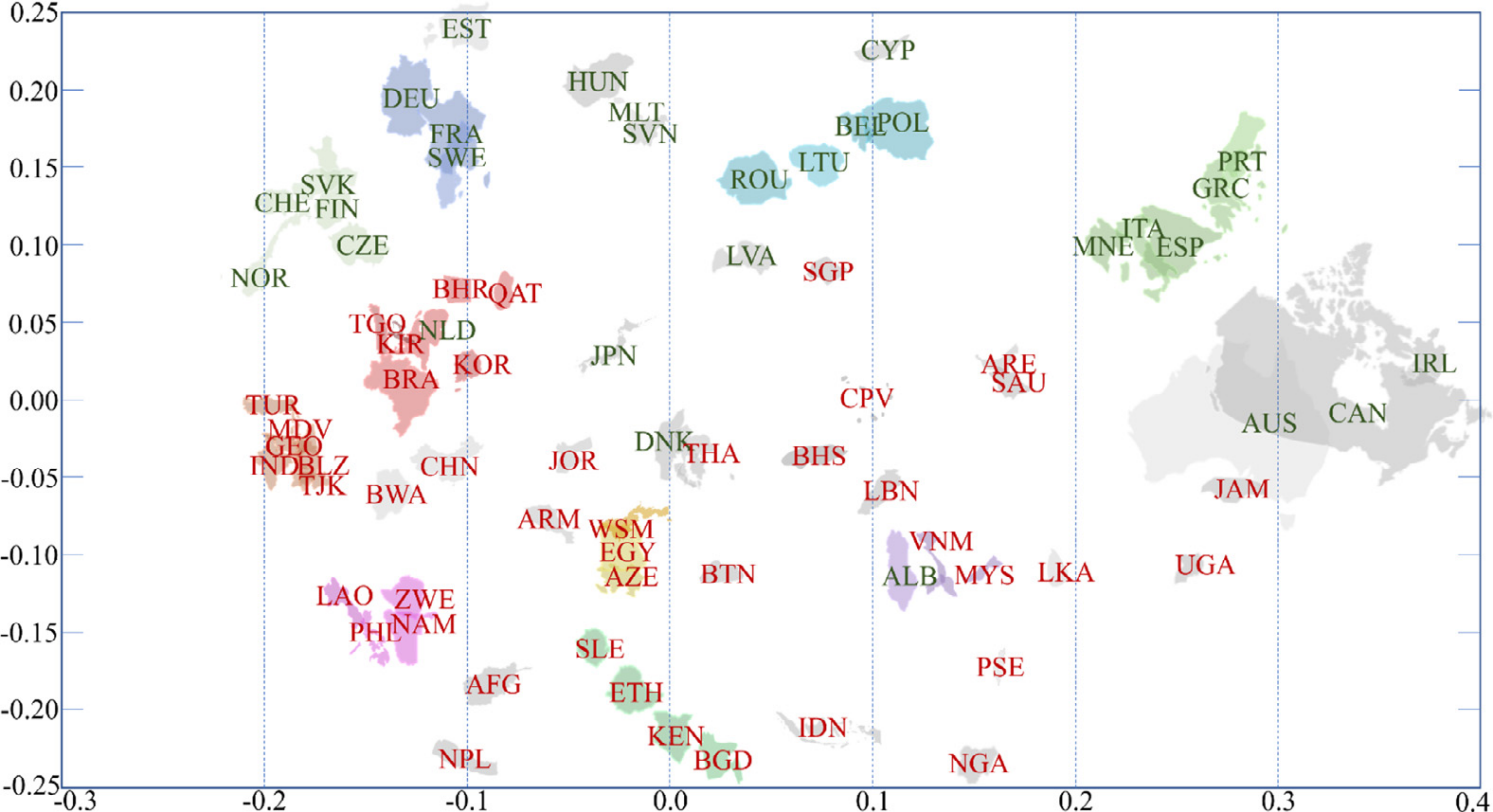
Similarity-based 2D visualization of countries around the world based on published VNRs.

Word pairs that are found in at least five documents and have a tf-idf value of equal or more than 0.6 were analyzed. The selection criteria depend on the scope of the analysis and the structure of the documents, therefore it should be determined by the analyst. The authors suggest to utilize criteria based on the tf-idf values of the analysed word pairs. The results of the analysis were validated by grouping the countries shown in Fig. 3. (and summarized in Fig. 4) based on the authors' expertise. Fig. 3. shows that there are countries in a group that has either a geographical similarity (common border) or an actual level of development. In Fig. 4. the shapes of the countries in the same group are marked with the same color. The color of the country code letters indicates their level of development (green: developed countries, red: developing countries). The summary results obtained support the expected groups of countries according to the sustainable development goals [16]. This result confirms that countries around the world can be grouped based on their VNR documents [16] with the proposed *MUNCoDA* method that is proven to be applicable to explore the relationships between documents in the same subject area.

The Matlab code of the dendrogram used for document comparison is the following:

**Table:** 

**%% clustering based on degree correlation Country**
CK=1-L_sim; %L_sim contains the similarity measures of the M documents, so it is an *M x M* matrix.
ZCK = linkage(CK,'complete');
figure (3)
[H,T,outperm_ck]= dendrogram(ZCK,0,'Orientation','left','Labels',listcountries);

The Matlab code of the multidimensional scaling based visualization of the similarities of the countries is the following:

**Table:** 

**%% Similarity-based 2D visualization of the documents**
figure (4)
clf
Y = mdscale(L_sim,2); %,'Start','random','criterion','sammon'
plot(Y(:,1),Y(:,2),'w.')
hold on
text(Y(:,1),Y(:,2),listcountries,'Fontsize',8);

## Conclusions

The multilayer network-based comparative analysis of documents (MUNCoDA) method has been developed to standardize and analyze valuable inconsistent textual information in documents dealing with the same subject area. The proposed method creates a multiplex network based on informative word pairs of documents, in which the edges represent the relevant relationships and can be weighted based on the information measures used in text mining. Networks can be analyzed on their own (per document) so that the relationships between topics and word pairs can be identified and visualized. Word pairs can be compared based on their node similarity and centrality measures. In the multiplex network, relationships can be classified based on edge overlap and layer similarity measures. Document exploration is based on an automated KNIME workflow and MATLAB/Octave code that calculates the informative word-pairs, generates the network and evaluates the similarities of the words and the documents.

## Declaration of Competing Interest

The authors declare that they have no known competing financial interests or personal relationships that could have appeared to influence the work reported in this paper.
